# *In Silico* and *In Vivo* Studies of β-Sitosterol Nanoparticles as a Potential Therapy for Isoprenaline-Induced Cognitive Impairment in Myocardial Infarction, Targeting Myeloperoxidase

**DOI:** 10.3390/ph17081093

**Published:** 2024-08-21

**Authors:** Partha Saradhi Tallapalli, Yennam Dastagiri Reddy, Deepak A. Yaraguppi, Surya Prabha Matangi, Ranadheer Reddy Challa, Bhaskar Vallamkonda, Sheikh F. Ahmad, Haneen A. Al-Mazroua, Mithun Rudrapal, Prasanth Dintakurthi Sree Naga Bala Krishna, Praveen Kumar Pasala

**Affiliations:** 1 Department of Pharmacology, Santhiram College of Pharmacy, JNTUA, Nandyal 518112, Andhra Pradesh, India; saradhitallapalli@gmail.com (P.S.T.); dastu1984@gmail.com (Y.D.R.); 2 Department of Biotechnology, KLE Technological University, Hubli 580020, Karnataka, India; deepak.yaraguppi@gmail.com; 3 Department of Pharmaceutics, School of Biotechnology and Pharmaceutical Sciences, Vignan’s Foundation for Science, Technology & Research, Guntur 522201, Andhra Pradesh, India; prabhalsurya@gmail.com (S.P.M.); rsmrpal@gmail.com (M.R.); 4 Department of Formulation and Development, Quotient Sciences, 3080 McCann Farm Dr, Garnet Valley, PA 19060, USA; ranadheerrc@gmail.com; 5 Department of Pharmaceutical Analysis, Odin Pharmaceutical LLC, Somerset, NJ 08873, USA; bhaskar718@gmail.com; 6 Department of Pharmacology and Toxicology, College of Pharmacy, King Saud University, Riyadh 11451, Saudi Arabiahalmazroua@ksu.edu.sa (H.A.A.-M.); 7 School of Pharmacy & Technology Management, SVKM’s Narsee Monjee Institute of Management Studies (NMIMS), Polepally SEZ, TSIIC, Jadcherla 509301, Andhra Pradesh, India; 8 Department of Pharmacology, Raghavendra Institute of Pharmaceutical Education and Research, JNTUA, Anantapuramu 515721, Andhra Pradesh, India

**Keywords:** β-sitosterol nanoparticle, myeloperoxidase, cognitive impairment, myocardial infarction, molecular simulation

## Abstract

Objective: This study aimed to compare the effects of β-sitosterol nanoparticles (BETNs) and β-sitosterol (BET) on cognitive impairment, oxidative stress, and inflammation in a myocardial infarction (MI) rat model using in silico and in vivo methods. Methods: β-Sitosterol (BET) and myeloperoxidase (MPO) ligand-receptor binding affinities were evaluated using Autodock Vina for docking and Gromacs for dynamics simulations. BET nanoparticles, prepared via solvent evaporation, had their size confirmed by a nanoparticle analyzer. ISO-induced cognitive impairment in rats was assessed through Morris water maze and Cook’s pole climbing tests. Oxidative stress, inflammation, and cardiac injury were evaluated by measuring GSH, SOD, MDA, MPO, CkMB, LDH, lipid profiles, and ECGs. Histopathology of the CA1 hippocampus and myocardial tissue was performed using H&E staining. Results: In silico analyses revealed strong binding affinities between BET and MPO, suggesting BET’s potential anti-inflammatory effect. BETN (119.6 ± 42.6 nm; PDI: 0.809) significantly improved MI-induced cognitive dysfunction in rats (*p* < 0.001 ***), increased hippocampal GSH (*p* < 0.01 **) and SOD (*p* < 0.01 **) levels, and decreased hippocampal MDA (*p* < 0.05 *) and MPO levels (*p* < 0.01 **). BETNs also elevated cardiac GSH (*p* < 0.01 **) and SOD (*p* < 0.01 **) levels and reduced cardiac MPO (*p* < 0.01 **), CkMB (*p* < 0.001 **) and LDH (*p* < 0.001 **) levels. It restored lipid profiles, normalized ECG patterns, and improved histology in the hippocampal CA1 region and myocardium. Conclusions: Compared with BET treatment, BETNs were more effective in improving cognitive impairment, oxidative damage, and inflammation in MI rats, suggesting its potential in treating cognitive dysfunction and associated pathological changes in MI.

## 1. Introduction

Cardiovascular diseases, such as myocardial infarction (MI), are a prominent contributor to public health problems in both developed and developing countries [1]. After myocardial infarction (MI), blood flow to the heart decreases, which is subsequently accompanied by an increase in reactive oxygen species (ROS) [2]. ROS are extremely reactive and harmful and can cause damage to proteins, lipids, and DNA [2,3]. In addition, heart disease leads not only to a reduced blood supply to the heart, but also to reduced blood flow to other organs, including the brain, which can lead to cognitive dysfunction [4,5,6,7,8]. Inflammation plays a crucial role in the pathogenesis of MI and associated post-MI complications, such as B. cognitive impairments. Myeloperoxidase (MPO), an important inflammatory biomarker associated with various diseases, such as ischemic heart disease and acute coronary syndrome, has gained attention as a potential biomarker for myocardial infarction and cognitive decline [9]. Furthermore, inflammatory biomarkers, such as MPO, particularly those related to cognitive aging, especially executive function, are increased in patients with Alzheimer’s dementia [10,11,12].

The administration of the beta-receptor agonist isoproterenol (ISO) was performed to observe the effects of acute sympathetic stress in a clinical setting. ISO administration consistently causes inflammation by stimulating the synthesis of cytokines, which leads to heart fibrosis [13], which can progress to left ventricular hypertrophy and dilatation [14]. This, in turn, results in reduced exploratory activity, reduced exploiter behavior [15], depressive-like behavior [16], and cognitive impairment in young, adult, male rats.

The human diet incorporates naturally occurring phytosterols, such as BET, which have cardiovascular advantages. Due to its antioxidative, anti-inflammatory, and neuroprotective properties, BET can be utilized as a preventive treatment for cognitive dysfunction and dementia in individuals with heart failure (HF). Many scientific reports have shown that it has anticancer [17], antidiabetic [18], antiviral [19], antimicrobial [20], nematic [21], immunomodulatory [22], antihyperlipidemic [23], and antiosteoporotic [24] properties. The clinical utility of BET is limited due to its poor bioavailability and susceptibility to metabolic degradation [25,26]. As a result, there is a low level of both systemic absorption and concentration at the target site. As a result, there is minimal absorption of the substance into the bloodstream, and the concentration at the intended location is also low. Therefore, we need innovative delivery systems that can overcome metabolic blockades and ensure the stability of these treatments to enhance their bioavailability for the treatment of heart failure-induced cognitive impairment. Recent advancements in nanoscience and nanotechnology have been developed to address these restrictions, resulting in the creation of novel medication delivery methods [27].

Molecular docking and molecular simulations are key techniques in the field of computational chemistry and structural biology. They play a crucial role in drug development and in gaining insights into molecular interactions [28]. Molecular docking is a technique for predicting how molecules bind with high specificity, which helps in the design of potent drugs [29]. Molecular simulations, such as molecular dynamics simulations, provide a dynamic description of molecular processes, stability, and interactions, and enable the detailed study of biological systems and their influence by mutations. These approaches complement each other and together provide a cost-effective and efficient alternative to experiments. Therefore, they will revolutionize the future of precision medicine and biotechnology by improving our understanding of molecular systems and enabling the development of novel therapeutics and materials [30]. The current study used *in silico* approaches to evaluate the activity of BET with myeloperoxidase (MPO). The accuracy of these computer predictions was confirmed by subsequent experimental studies. We synthesized BETNs and evaluated their efficacy by assessing cognitive function, the hippocampus, and cardiac antioxidants in rats with ISO-induced myocardial infarction. The results were further validated by in silico studies.

## 2. Results

### 2.1. Molecular Docking

**Figure 1:**
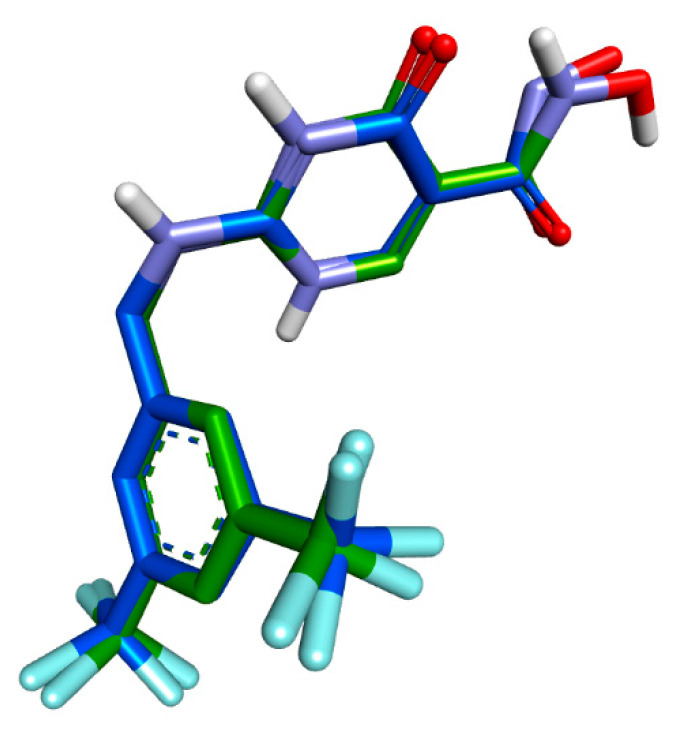
Validation of the docking algorithm by redocking the native inhibitor (2-{[3,5-bis(trifluoromethyl)benzyl]amino}-n-hydroxy-6-oxo-1,6-dihydropyrimidine-5-carboxamide) (CCl) on the target MPO (PDB ID: 4C1M). Blue—native crystallized position of CCl; green: docked position of CCl.

Computational chemistry has gained significance in medicine, especially in drug design, where computational methods study ligand–receptor interactions using various docking techniques. These techniques depend on the interaction between a rigid active site and a ligand molecule binding to the pocket. To validate one technique, we redocked a cocrystallized ligand into its binding site’s active pocket. Figure 1 demonstrates the docking process’s effectiveness and reliability, with an RMSD of 0.935926 Å between the redocked and crystallized poses.

**Figure 2:**
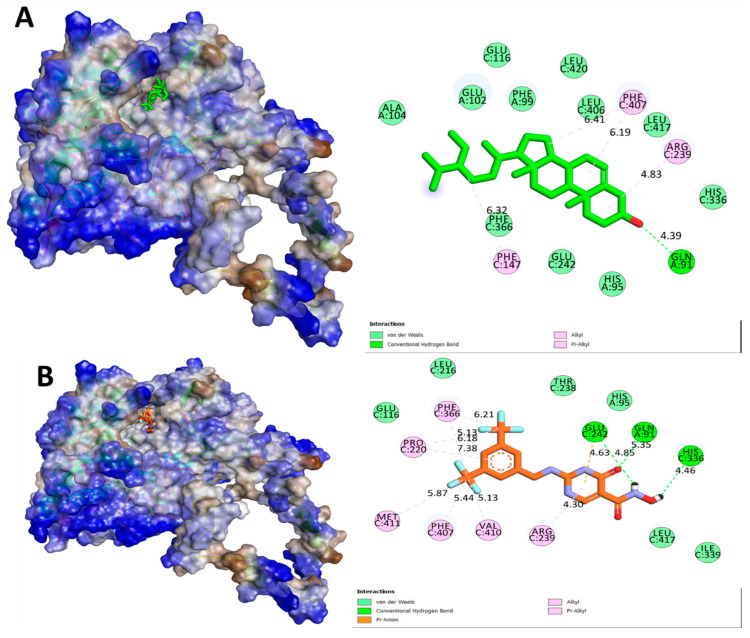
Molecular surface view and 2D visualization of the interaction between the selected ligand and MPO using AutoDock Vina and Biovia Discovery Studio. (**A**) BET and (**B**) CCl.

**Figure 3:**
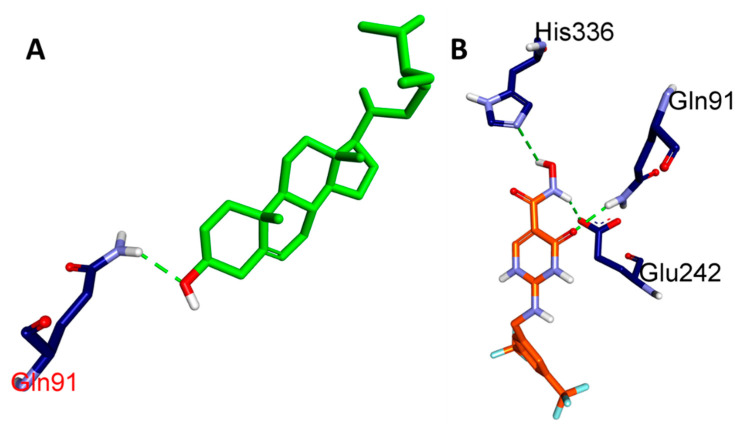
Three-dimensional view of the hydrogen bond interactions of selected ligands with MPO: (**A**) BET and (**B**) CCl.

**Figure 4:**
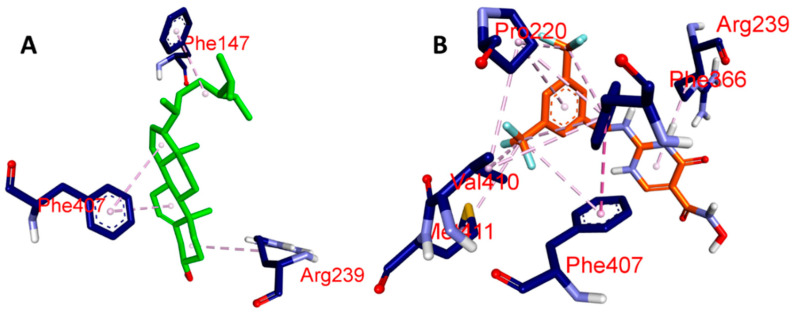
Three-dimensional view of the hydrophobic bond interactions of selected ligands with MPO: (**A**) BET and (**B**) CCl.

**Figure 5:**
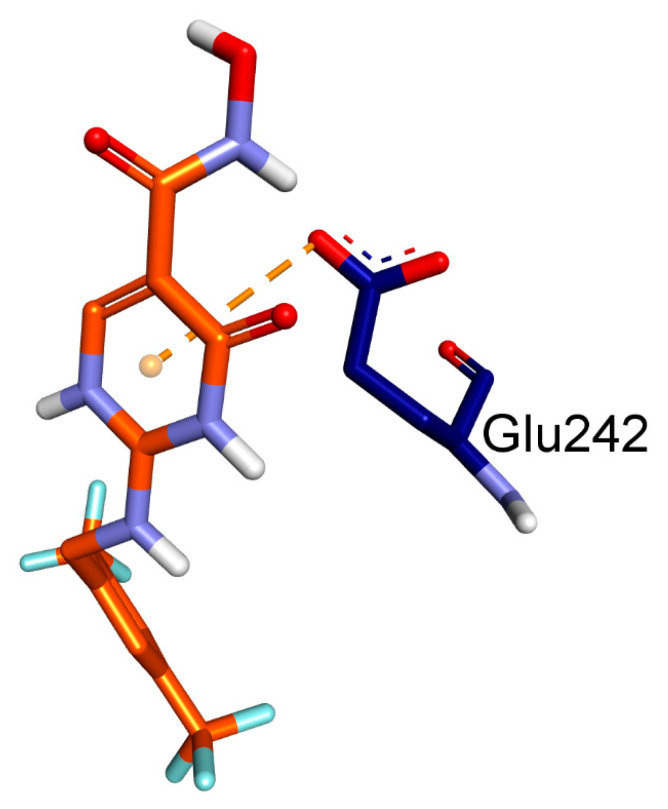
Three-dimensional view of the electrostatic interactions between CCl and MPO via Glu242.

In the present study, we aimed to investigate the binding affinities and interactions of two ligands, namely, BET and CCl, with MPO. We used molecular docking studies to understand the atomistic details of the complexation process and to further characterize the behavior of these two ligands with the protein. The free energy change (ΔG) for the formation of the ligand-MPO complex was calculated, and the key interacting residues were identified based on the free energy decomposition per residue. We found that the BET and CCl ligands showed exceptionally high binding affinities toward MPO, with calculated ΔG values of −7.1 and −7.7 kcal/mol, respectively, suggesting that these ligands form stable complexes with MPO (Table 1). The GLN A:91, PHE A:147, ARG A:239, and PHE A:407 residues of MPO formed a prominent H-bond network with BET, which was mainly responsible for the high binding affinity. When CCl was complexed with MPO, HIS A:91, GLU A:242, and HIS A:336 were found to form the main H-bonding network. However, we also found that the MPO residues involved in hydrophobic and hydrogen bonding interactions with CCl included ARG A:239, VAL A:410, PHE A:407, MET A:411, PRO A:220, and PHE A:366. Based on the above findings, it can be strongly suspected that both BET and CCl are extremely potent ligands of their respective proteins with high binding affinities. In summary, our study provides evidence that specific MPO residues are critical for BET and CCl binding and may be exploited for the use of these ligands for therapeutic purposes in myocardial ischemia (Figure 2, Figure 3, Figure 4 and Figure 5).

### 2.2. Molecular Dynamics

All-atom molecular dynamics simulations can provide invaluable information about protein structural dynamics and ligand binding interactions. This method provides a detailed representation of chemical systems and has dramatically improved the provision for computational drug design. Here, we used the binding of the target protein to model dynamic changes via molecular dynamics (MD) simulations. Similarly, several parameters, such as the root mean square deviation (RMSD), root mean square fluctuation (RMSF), radius of gyration (Rg), solvent accessible surface area (SASA), and interhydrogen bonding, were calculated for the protein alone and its protein–ligand complex.

#### 2.2.1. Root Mean Square Deviation (RMSD)

**Figure 6:**
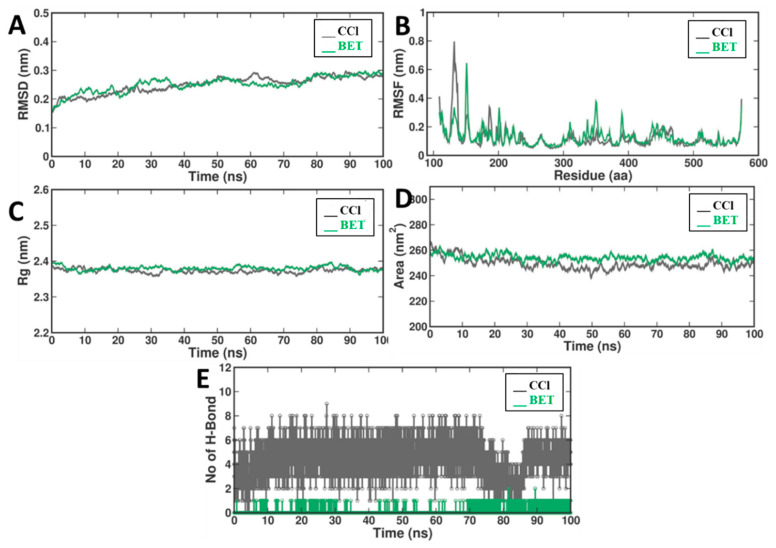
Molecular simulation plots of BET and CCl with 4C1M. (**A**) RMSD plot; (**B**) RMSF plot; (**C**) Rg plot; (**D**) SASA plot; and (**E**) number of hydrogen bonds over the simulation time.

The RMSD is of utmost importance for evaluating the stability of a protein–ligand complex using molecular dynamics (MD) simulations. This stability is also reflected in the RMSD profiles of the two ligand systems, CCl and BET, shown in Figure 6A, where both complexes quickly reach equilibrium after the first 10 ns. The initial position in the binding pocket was energetically favorable and equilibrated within the first few ns. The RMSD values for both systems were quite stable in the last 100 ns of the simulation, with an RMSD of ~0.25 ± 0.02 nm for both the CCl and BET ligands. This uniformity implies that the conformation of the complexes did not change significantly during the simulation, apart from the fluctuations that occurred in the molecular simulation. Taken together, these results suggest that the stable conformation is solely due to the strong binding of the protein to the ligands and provides suitable docked complexes for further analysis. Furthermore, the BET ligand did not have a significant impact on the stability of the protein structure compared to that of the reference system, as shown by a comparison of the RMSD values. Therefore, the stability of the binding of the BET ligand to the binding pocket of the target protein not only highlights its good design, but also indicates that it may be a useful lead structure.

#### 2.2.2. Root Mean Square Fluctuation (RMSF)

RMSF analysis is very useful for studying the flexibility of individual residues of a protein during MD simulations. This measure can be used to visualize the movement of each residue during simulations and identify regions of the protein structure that are more rigid or flexible. The secondary structure data (α-helices and β-sheets) show small RMSF values, suggesting that these regions are rigid (Figure 6B). Areas with a higher RMSF, such as loops and terminals, tend to be more flexible. The RMSF values for the CCl and BET ligand complexes in this study are shown in Figure 6B. The average RMSF was 0.12 ± 0.08 nm for the CCl complex and 0.12 ± 0.06 nm for the BET complex. This finding suggests that the movement of residues in the protein is consistent with the NMR data. Similar RMSF values indicate that no significant fluctuations in protein dynamics occur upon binding to the CCl or BET ligands. Surprisingly, the global dynamics remained almost unchanged upon the binding of both ligands, suggesting that the protein flexibility of the residues was not significantly restricted. This indicates that the protein has a native structure and functions upon ligand binding. Conserved RMSF values indicate that the interactions are stable and do not significantly distort the flexibility of the native protein. These ligands are important because they appear to stabilize the protein, and they may represent ligands that can provide structural support to the protein upon binding, which is important for its functional viability.

#### 2.2.3. Radius of Gyration (Rg)

Among the MD simulations, the radius of gyration (Rg) is one of the most important parameters because the compactness and stability of proteins are clear from a structural point of view. It defines the distribution of the protein mass around its center of mass and describes the changes in the stability and folding of the protein–ligand complexes during the simulation. The Rg values for the CCl and BET ligand complexes are shown in Figure 6C. The average Rg of the BET complex (2.35 ± 0.007 nm) was slightly lower than that of the CCl complex (2.37 ± 0.007 nm). This suggests that, in the 100 ns simulation, both complexes remained tightly packed. The pattern of small Rg values at which the BET ligand binds in the presence and absence of CCl, suggests that the BET ligand containing CCl does not significantly alter the compactness of CCl. This is the hallmark of a well-functioning interaction, in which the protein sticks to its resting form in the presence of the ligand. There are very few changes in the Rg values, suggesting that these are fairly stable complexes with no BET ligand-mediated structural changes in the protein. Despite their simplicity, these findings are fundamental to understanding the fundamental biophysical properties of protein–ligand interactions. Here, they showed that BET ligands can bind proteins in a way that does not compromise their overall compactness. The formation of stable interactions of this type is of particular importance in drug design, as it avoids a ligand-induced destabilization of the protein’s native conformation and thus preserves its biological function.

#### 2.2.4. Solvent Accessible Surface Area (SASA)

The solvent accessible surface area (SASA) is an important parameter for determining the extent of exposure of a protein molecule to a solvent. This shows how the protein interacts with water (surface properties) and where the ligands bind to the protein. SASA values are related to protein folding and can be used as a first approximation to determine the residues that may be involved in protein function and interaction. The SASA values for the CCl and BET ligand complexes calculated from 100 ns simulations are shown in Figure 6D. The SASA values, which were stable during the simulation, were consistent for both complexes as follows: CCl, 248.33 ± 3.9 nm^2^; BET, 253.92 ± 3.5 nm^2^. These relatively stable SASA values indicate that the solvent accessibility of the protein remained almost unchanged after binding of the BET ligand. For the BET complex, a slight increase in the SASA reflects a marginally larger surface area exposed due to binding interactions and no conformational changes. The time-averaged SASA values remained constant throughout the simulation, indicating that the protein–ligand interaction was stable and that the protein surface residues were not significantly disturbed by ligand binding. This stability is required to maintain the protein’s functional sites and to bind to other molecules in the solvent environment.

#### 2.2.5. Interhydrogen Bonds

However, to gain a comprehensive understanding of protein–ligand stability, the formation and duration of hydrogen bonds must be taken into account. In this study, we focused on the hydrogen bonds between BET groups over time, as shown in Figure 6E, showing that the docked complex was extremely stable throughout the simulation. The presence of hydrogen bonds (1–9) leads to interactions with the BET complex, and therefore, any stability is associated with their presence. The range of these hydrogen bonds is more than one but not more than nine in total, as the simulations show. The consistent formation of these hydrogen bonds highlights their essential role in stabilizing the protein–ligand complex. The graph represents the number of hydrogen bonds at each point in time and shows that the number changes within the specified range but never drops below one. This suggests that the hydrogen bonds are well defined and contribute significantly to the overall stability of the complex. Furthermore, the upper end of the range with up to nine hydrogen bonds suggests that a significant number of protein–ligand interactions are both strong and long lasting, which may help explain the increased stability. In summary, our results highlight the crucial role of hydrogen bonds in maintaining protein–ligand interactions, particularly in the context of BET complexes.

#### 2.2.6. MM-PBSA

We evaluated the binding strengths of CCl and BET within the summer energy protein and examined how well they bound together using Table 2 for the inhibitors calculated with the MM-PBSA technique. By conducting a steady simulation, we were able to determine the individual contributions of each residue to the interaction energy. The data reveal that CCl possesses a van der Waals energy of −112.771 ± 23.244 kJ/mol, an electrostatic energy of −146.272 ± 27.497 kJ/mol, a polar solvation energy of 253.003 ± 70.776 kJ/mol, and a binding energy of −22.793 ± 30.727 kJ/mol. Moreover, BET exhibited a van der Waals energy of −180.276 ± 9.119 kJ/mol, an electrostatic energy of 1.010 ± 5.273 kJ/mol, a polar solvation energy of 61.251 ± 15.562 kJ/mol, and a binding energy of −140.699 ± 13.108 kJ/mol.

### 2.3. Characterization of BETNs

**Figure 7:**
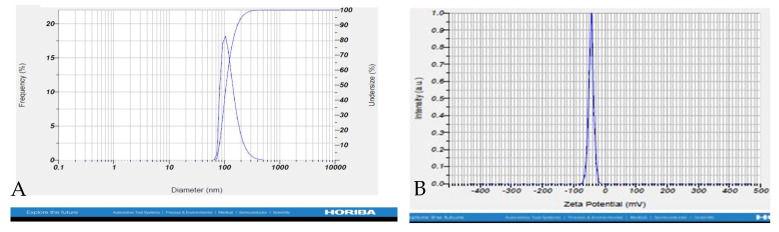
Characterization of BETNs: (**A**) particle size analysis and (**B**) particle charge analysis.

The prepared BETNs were found to have a particle size of 119.6 ± 42.6 nm with a polydispersity index of 0.809. The prepared nanoparticles exhibited a zeta potential of −43.8 mV, indicating their stability. We determined that the developed nanoparticles had an appropriate size and were suitable for conducting biological experiments (Figure 7).

### 2.4. Effect of BETNs on ECG in ISO-Treated Rats

**Figure 8:**
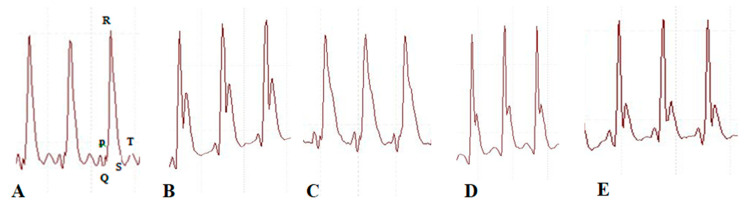
Effect of BETNs on the electrocardiograms of ISO-treated rats: (**A**) group I, (**B**) group II, (**C**) group III, (**D**) group IV, and (**E**) group V. P wave in an ECG complex indicates atrial depolarization. The QRS is responsible for ventricular depolarization and the T wave is ventricular repolarization.

**Figure 9:**
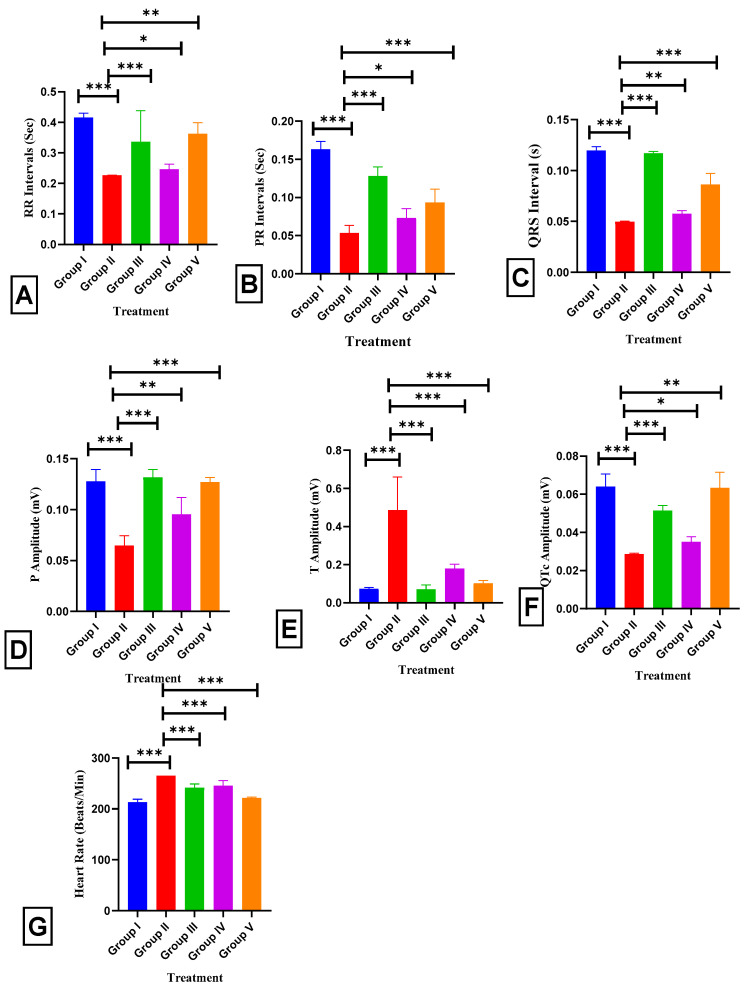
Effect of BETNs on the electrocardiograms of ISO-treated rats: (**A**) RR intervals, (**B**) PR intervals, (**C**) QRS intervals, (**D**) P amplitude, (**E**) T amplitude, (**F**) QTc amplitude, and (**G**) heart rate. *p* < 0.05 *, *p* < 0.01 **, and *p* < 0.001 *** Significance comparison treatment group vs. Disease group.

We recorded an electrocardiogram (Figure 8 and Figure 9) to evaluate the efficacy of BETNs on ISO-induced alterations in the electrical activity of the heart. When the rats were administered ISO, the RR intervals were in the range of 0.42 ± 0.011 to 0.23 ± 0.001 (*p* < 0.001 **), the PR intervals were in the range of 0.16 ± 0.013 to 0.05 ± 0.022 (*p* < 0.001 ***), the QRS intervals were in the range of 0.12 ± 0.005 to 0.05 ± 0.001 (*p* < 0.001 ***), the P amplitudes were in the range of 0.13 ± 0.02 to 0.06 ± 0.02 (*p* < 0.001 ***), the QTc amplitudes were in the range of 0.06 ± 0.01 to 0.03 ± 0.002 (*p* < 0.001 ***), and the heart rates were in the range of 213.83 ± 0.2 to 265 ± 0.16 (*p* < 0.001 ***). All of these findings are signs of myocardial infarction. Treatment with 40 mg/kg b.wt. BETNs before the study improved heart function, as indicated by the RR interval (0.36 ± 0.03; *p* < 0.01 **), PR interval (0.09 ± 0.029; *p* < 0.001), QRS interval (0.09 ± 0.019; *p* < 0.001), P amplitude (0.13 ± 0.006; *p* < 0.001), QTc amplitude (0.06 ± 0.017; *p* < 0.01 **), decreasing T amplitude (0.1 ± 0.023; *p* < 0.001 ***), and heart rate (221.5 ± 0.05; *p* < 0.001 ***).

### 2.5. Effect of BETNs on Cardiac Parameters and Lipid Profiles in ISO-Treated Rats

**Figure 10:**
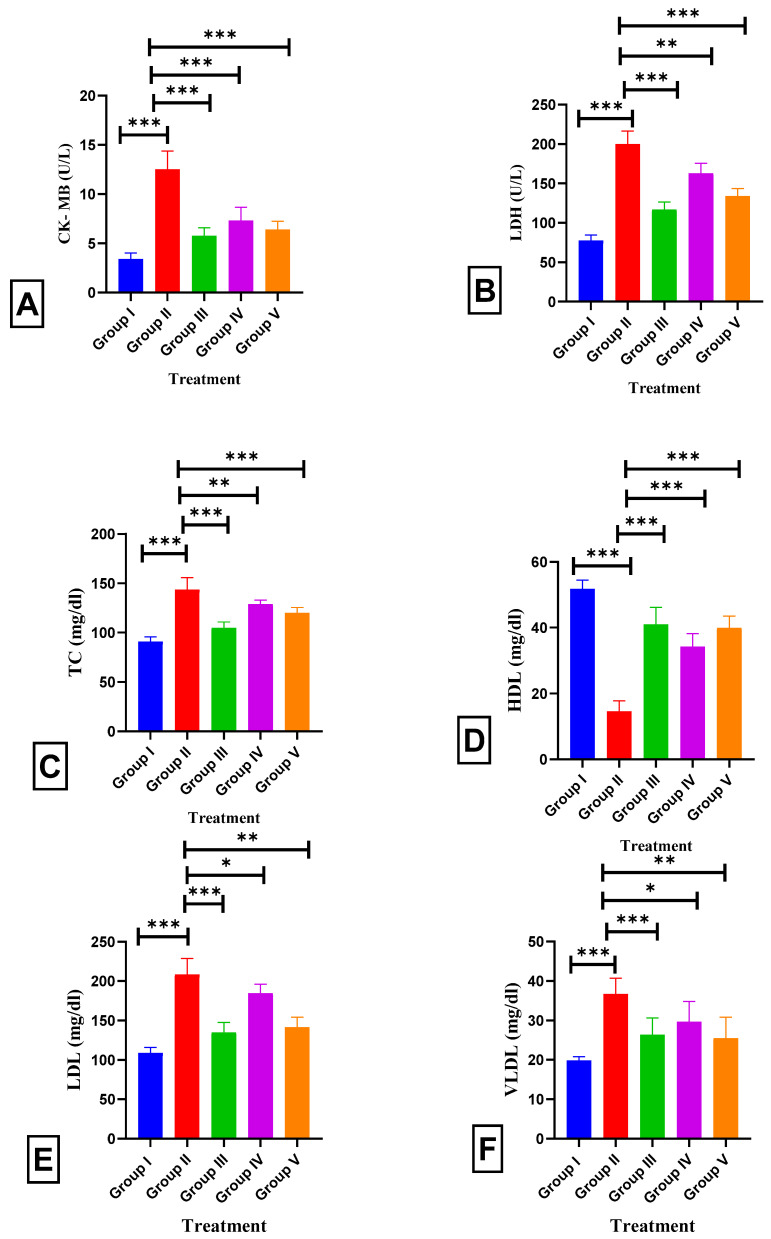
Effect of BETNs on the lipid profiles of ISO-treated rats: (**A**) CkMB, (**B**) LDH, (**C**) TC, (**D**) HDL, (**E**) LDL, and (**F**) VLDL. *p* < 0.05 *, *p* < 0.01 **, and *p* < 0.001 *** Significance comparison treatment group vs. Disease group.

Figure 10 summarizes the serum lipid profiles and cardiac parameters of ISO-treated rats treated with BETNs. The rats that were administered ISO had greater amounts of TC (90.9 ± 0.2 to 143.8 ± 0.4; *p* < 0.001), LDL (108.8 ± 0.3 to 14.6 ± 0.4; *p* < 0.001), and VLDL (19.8 ± 0.1 to 36.67 ± 0.3; *p* < 0.001); lower amounts of HDL (51.8 ± 0.1 to 16.2 ± 0.47; *p* < 0.001); and greater amounts of Ck-MB (3.4 ± 0.167 to 12.5 ± 0.2; *p* < 0.001) and LDH (77.5 ± 0.4 to 200.17 ± 0.58; *p* < 0.001). Before administering BETNs (40 mg/kg b.wt.) to rats, serum TC (119.8 ± 0.2; *p* < 0.001 ***), LDL (141.33 ± 0.53; *p* < 0.01 ***), and VLDL (25.5 ± 0.5; *p* < 0.01 ***) levels decreased significantly, while HDL levels increased significantly (39.9 ± 0.2; *p* < 0.001 ***), and the concentrations of the heart markers Ck-MB (6.4 ± 0.1; *p* < 0.01) and LDH (134 ± 0.4; *p* < 0.001) decreased significantly compared to those in rats treated with ISO and BET (40 mg/kg). The same outcome was observed in the ATV group.

### 2.6. Effect of BETNs on Cognitive Function

#### 2.6.1. MWM Test

**Figure 11:**
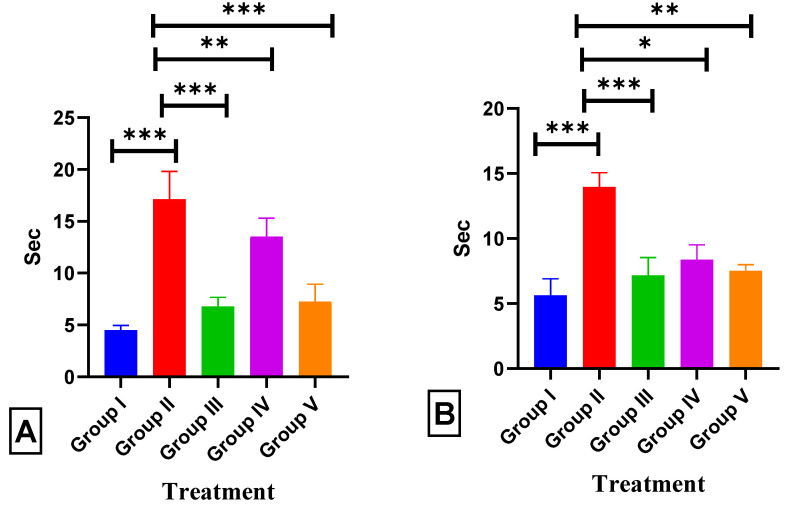
Effect of BETNs on cognitive function in ISO-treated rats: (**A**) MWM test and (**B**) CPC test. *p* < 0.05 *, *p* < 0.01 **, and *p* < 0.001 *** Significance comparison treatment group vs. Disease group.

We conducted an experiment to determine the impact of BETNs on long-term and spatial memory using the Morris water maze (MWM) test. All groups, except for the ISO group, exhibited a reduction in the time it took for the rats to locate the escape platform during the experimental period. ISO-treated rats, on the other hand, demonstrated a significantly prolonged escape latency of 17.13 ± 0.33 (*p* < 0.001 ***) compared to that of normal rats. These findings indicate that ISO causes long-term and spatial memory impairments. In contrast, the administration of BETNs (40 mg/kg B.wt.) had a decrease in escape latency of 7.25 ± 0.3 (*p* < 0.001 ***) compared to that of the BET-treated and ISO-treated rats (Figure 11A).

#### 2.6.2. Pole Climb Test

The pole climb test was used to evaluate the escape latency (conditional avoidance) of each rat during the learning and retention phases. During the initial acquisition phase, no significant differences were observed in the escape latency time (ELT) between ISO-treated rats and normal rats. However, on the 21st day, a substantial difference was evident between the positive control group’s ELT (13.97 ± 0.14; *p* < 0.001 ***) and the normal group’s ELT (5.62 ± 0.2). In contrast, when BETNs (40 mg/kg B.wt.) were administered to the rats, their ELT was lower (7.5 ± 0.09; *p* < 0.01 **) than that of the rats that were administered ISO and BET (as depicted in Figure 11B).

### 2.7. Effects of BETNs on Hippocampal and Cardiac Biochemical Parameters in ISO-Treated Rats

**Figure 12:**
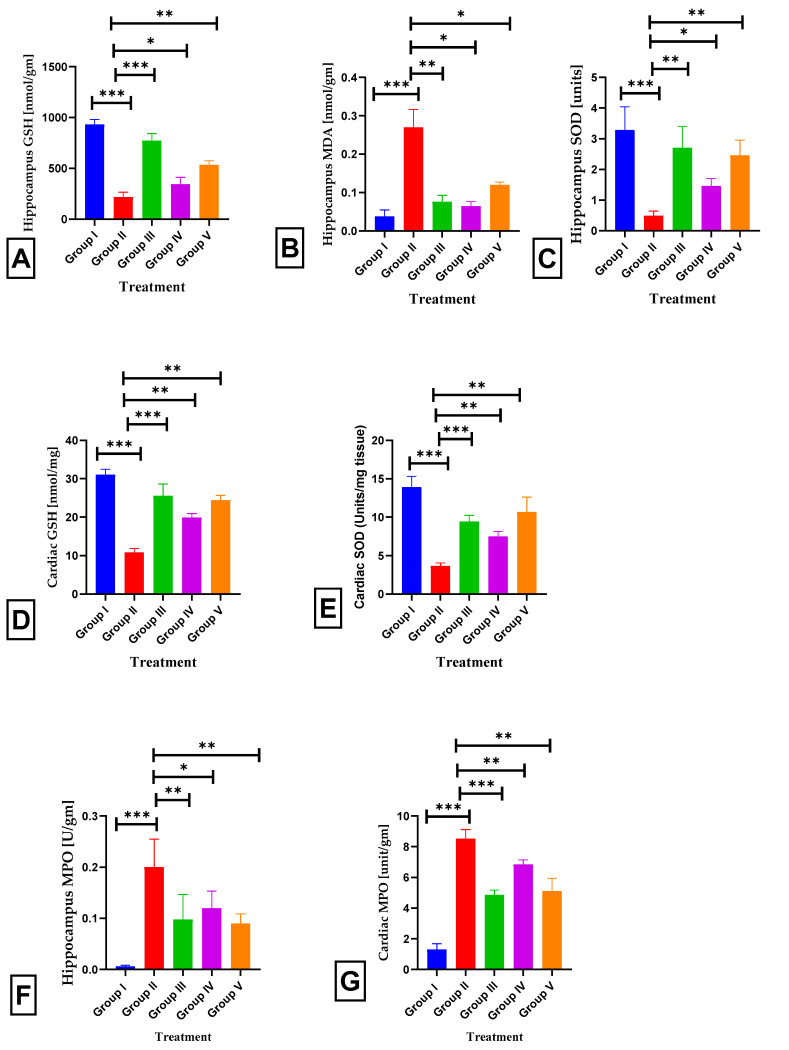
Effect of BETNs on tissue parameters. (**A**) Hippocampal GSH. (**B**) Hippocampal MDA. (**C**) Hippocampal SOD. (**D**) Cardiac GSH. (**E**) Cardiac SOD. (**F**) Hippocampal MPO. (**G**) Cardiac MPO. *p* < 0.05 *, *p* < 0.01 **, and *p* < 0.001 *** Significance comparison treatment group vs. Disease group.

ISO injection resulted in a substantial decrease in hippocampal GSH activity (218.17 ± 1.6, *p* < 0.001 ***) compared to that in normal rats (930.83 ± 0.8). When 40 mg/kg of body-weight BETNs was administered to rats, it significantly increased GSH activity (535.67 ± 0.81, *p* < 0.01 **) compared to that in rats that were administered ISO or BET (344.33 ± 1.8, *p* < 0.05 *) (Figure 12A). ISO injection resulted in a substantial increase in hippocampal MDA activity (0.27 ± 0.045, *p* < 0.001 ***) compared to that in normal rats (0.04 ± 0.042). However, the administration of BETNs at a dosage of 40 mg/kg body weight resulted in a substantial decrease in MDA activity (0.12 ± 0.01, *p* < 0.05 *) compared to that in both the ISO rats and the rats treated with BET (0.07 ± 0.022, *p* < 0.05 *) (Figure 12B). ISO injection resulted in a substantial decrease in hippocampal SOD activity (0.27 ± 0.045, *p* < 0.001 ***) compared to that in normal rats (0.04 ± 0.042). However, following treatment with BETNs (40 mg/kg b.wt.), SOD activity (0.12 ± 0.01, *p* < 0.01 **) was significantly greater in the ISO-treated rats than in the BET-treated rats (0.07 ± 0.022, *p* < 0.05 *) (Figure 12C). ISO administration resulted in a significant decrease in cardiac GSH activity (12.3 ± 1.4, *p* < 0.001 ***) compared to that in normal rats (30.2 ± 1.6). However, the administration of BETNs at a dose of 40 mg/kg body weight resulted in a significant reduction in MPO activity (23.9 ± 0.03, *p* < 0.01 **) compared to both the ISO rats and the rats treated with BET (19.22 ± 0.048, *p* < 0.01 **) (Figure 12D). ISO administration resulted in a substantial decrease in cardiac SOD activity (3.66 ± 0.1, *p* < 0.001 ***) compared to that in normal rats (13.92 ± 0.18). However, the administration of BETNs at a dosage of 40 mg/kg body weight resulted in a significant increase in SOD activity (10.68 ± 0.2, *p* < 0.01 **) compared to both the ISO rats and the rats treated with BET (7.52 ± 0.12, *p* < 0.01 **) (Figure 12E). The administration of ISO significantly enhanced hippocampal MPO activity (0.2 ± 0.06, *p* < 0.001 ***) compared to that in normal rats (0.01 ± 0.014). However, the administration of BETNs at a dosage of 40 mg/kg body weight resulted in a substantial decrease in MPO activity (0.09 ± 0.03, *p* < 0.01 **) compared to both the ISO rats and the rats treated with BET (0.12 ± 0.048, *p* < 0.05 *) (Figure 12F). The administration of ISO resulted in a considerable increase in cardiac MPO activity (8.52 ± 0.1, *p* < 0.001 ***) compared to that in normal rats (1.3 ± 0.16). However, the administration of BETNs at a dose of 40 mg/kg body weight resulted in a significant reduction in MPO activity (5.1 ± 0.1, *p* < 0.01 **) compared to both the ISO rats and the rats treated with BET (6.84 ± 0.057, *p* < 0.01 **) (Figure 12G).

### 2.8. Histopathological Studies

**Figure 13:**
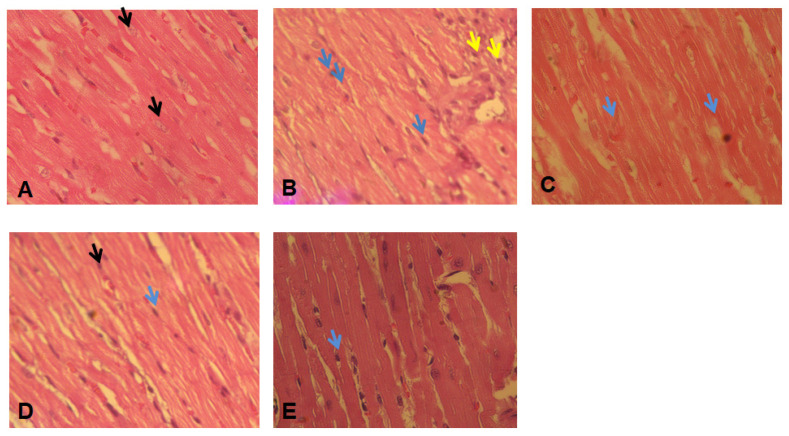
Myocardium histopathology: (**A**) The control group exhibited the standard histological architecture of cardiac myocytes, characterized by prominent nuclei (black arrow). (**B**) ISO treatment resulted in a marked degeneration of cardiac muscle fibers, extensive damage to cardiac myocytes (yellow arrow), disarrangement of nuclei, and marked inflammatory cell infiltration (blue arrow). (**C**) ISO + ATV treatment produced uniformly arranged myocardial fibers and myocytes with uniform nuclei and striations (blue arrow). (**D**) ISO + BET (40 mg/kg) treatment caused damage to cardiac myocytes (blue arrow) and a few cardiac myocytes with prominent nuclei (black arrow). (**E**) ISO + BETN (40 mg/kg) treatment resulted in compact, uniformly arranged myocardial fibers with uniformly and prominently placed nuclei (blue arrow).

Histopathological analysis of cardiac muscle fibers after ISO treatment revealed a marked degeneration of the cardiac muscle fibers (yellow arrow), disarrangement of the nuclei, and marked inflammatory cell infiltration (blue arrow) [31]. ATV therapy resulted in the formation of uniformly oriented cardiac fibers and myocytes with uniform nuclei and striations (shown by the blue arrow). The administration of BETNs (40 mg/kg) led to the formation of tightly packed cardiac fibers that were uniformly organized, with nuclei positioned uniformly and prominently (shown by the blue arrow), in comparison to BET therapy (Figure 13).

**Figure 14:**
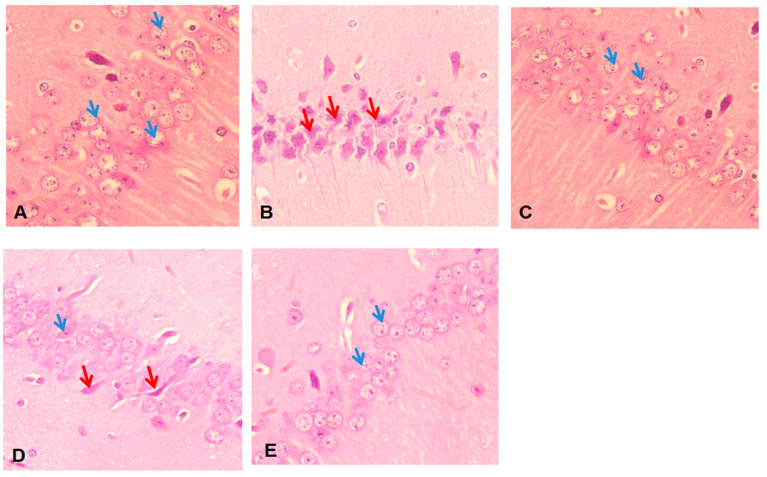
Hippocampal CA1 histopathology. (**A**) Normal rats: In the CA1 region, pyramidal neuron cell bodies are closely packed and arranged in 3 to 4 rows, appearing small with vesicular nuclei, prominent nucleoli, and scant cytoplasms (blue arrow). (**B**) In ISO-treated rats, the cell bodies of pyramidal neurons in the A1 region are disarranged and loosely packed, appearing dark, skinny, and with pyknotic nuclei (red arrow). (**C**) In ISO + ATV-treated rats, the cell bodies of pyramidal neurons are somewhat regularly arranged and loosely packed. (**D**) In ISO + BET (40 mg/kg)-treated rats, the granule cell bodies in the DG region are well defined and exhibit pyknotic nuclei (red arrow). (**E**) In ISO + BETN (40 mg/kg)-treated rats, the cell bodies of pyramidal neurons in the CA1 region are packed and prominent, arranged in 3 to 4 rows, with nuclei appearing prominent (blue arrow).

The histopathology of the hippocampus in normal rats exhibited no alterations, with the typical features of the CA1 region showing intact pyramidal neurons in the hippocampus region (Figure 14). In the CA1 region of normal rats, the cell bodies of pyramidal neurons are densely packed and organized into three to four rows. These cells appear as tiny and have nuclei that include vesicles, conspicuous nucleoli, and a modest amount of cytoplasm (shown by the blue arrow). The pyramidal neurons in the CA1 region of rats treated with ISO exhibited disorganized and sparsely distributed cell bodies. These neurons seem narrow, black, and have condensed nuclei, as indicated by the red arrow. The pyramidal neurons in rats treated with ATV exhibited a moderately organized and sparsely distributed arrangement of cell bodies. In rats treated with BETNs (40 mg/kg), the cell bodies of pyramidal neurons in the CA1 region are densely packed and easily noticeable. They are organized in three to four rows, and the nuclei are particularly apparent (shown by the blue arrow). The hippocampal CA1 region in the ISO-treated group of rats treated with 40 mg/kg BETNs exhibited considerably less damage than that in the ISO-treated group of rats treated with 40 mg/kg BET. These findings are consistent with earlier studies [32].

## 3. Discussion

The primary objective of this investigation was to delineate the therapeutic potential of BETNs for cognitive impairment associated with myocardial infarction (MI) by considering myeloperoxidase (MPO) a pivotal inflammatory biomarker. This research is particularly timely and relevant, as cognitive decline following MI is a significant clinical concern, with current treatment options being limited and not directly addressing the inflammatory component of the disease pathology [33]. Inflammation following MI is a critical driver of cognitive deficits, and MPO, a lysosomal enzyme released by activated leukocytes, has emerged as a significant contributor to oxidative stress and inflammatory responses in the post-MI context. By focusing on MPO, this study aligns with the emerging paradigm shift in the management of MI-associated cognitive deficits targeting the inflammatory cascade to halt or reverse neurological damage. BET nanoparticles (BETNs) offer a novel therapeutic modality, potentially improving the specificity of treatments designed to mitigate the cognitive sequelae of ischemic heart disease.

The comprehensive molecular docking analyses presented in our study revealed that BET and the control ligand (2-{[3,5-bis(trifluoromethyl)benzyl]amino}-n-hydroxy-6-oxo-1,6-dihydropyrimidine-5-carboxamide) (CCl) have high binding affinities for myeloperoxidase (MPO), with calculated free energy changes (ΔG) of −7.1 and −7.7 kcal/mol, respectively. These robust ΔG values strongly suggest the potential of both ligands to form stable complexes with MPO, an enzyme particularly involved in the inflammatory response associated with MI [34]. The establishment of these stable complexes may lead to attenuation of MPO activity, which could subsequently lead to a reduction in oxidative stress and inflammation, which are major contributors to myocardial infarction progression and post-MI cognitive deficits.

The stability of protein–ligand systems is one of the key parameters that can be explained by the root mean square deviation (RMSD) of our molecular dynamics simulation [35]. The low and stable RMSD values of both BET and CCl molecules over the simulation period supported good system stability [36]. Equilibrium was achieved within a period of 10 ns and maintained throughout the simulation period of 100 ns. This stability can be attributed to the fact that both complexes are conformationally stable, which is the primary property for the acceptability of these compounds from a therapeutic perspective when considered under in vivo physiological conditions [37].

The analysis of the RMSF results is of particular interest to clarify how ligand binding affects changes in protein flexibility. Root mean square fluctuation (RMSF) measures the amplitude of fluctuations that an individual residue experiences over the course of the simulation with respect to its reference position, providing an estimate of the local flexibility within the protein structure [38]. As shown in Figure 6, the concurrent RMSF values for both the BET and CCl complexes of MPO strongly preserve the protein flexibility patterns upon ligand binding. The almost complete lack of measurable flexibility differences suggests the stabilizing effect of the binding of these ligands, as the presence of the ligands in the complex does not lead to significant conformational changes within the protein MPO that could ultimately affect its functionality. Such flexibility is likely to play a role in maintaining the properties of the ligand-bound form of the enzyme, suggesting that BET and CCl may indeed function as modulators of MPO activity without disturbing their inherent dynamic properties.

The radius of gyration (Rg) determined from our molecular dynamics simulations serves as a crucial indicator of the structural integrity and compactness of the CCl and BET complexes [39]. The observed Rg values for both systems, an average of 2.37 ± 0.007 nm for CCl and 2.35 ± 0.007 nm for BET, indicate a high degree of structural stability conferred by the binding of BETNs to myeloperoxidase. This compactness suggests a densely arranged tertiary structure, characteristic of a protein–ligand complex that is less likely to be disrupted under physiological conditions.

Solvent accessible surface area (SASA) analysis provides information about how much of the protein is exposed to the solvent [40]. The primary influence here is the potential pharmacokinetic (PK) properties of a drug. Our SASA results show a very stable profile for both CCl and BET over the simulation time, such that binding of these ligands does not appear to cause any measured changes in conformational stability, which can be inferred from significant changes in the level of solvent exposure. The average SASA values for CCl and BET were 248.33 ± 3.9 nm^2^ and 253.92 ± 3.5 nm^2^, respectively, with completely consistent solvent accessibility profiles. This positive property potentially delays the influence of CCl and BET binding to myeloperoxidase (MPO) on the surface properties that could affect drug distribution and absorption and should contribute positively to the consistent pharmacokinetic properties.

Intermolecular hydrogen bonds are crucial for stabilizing the complex structure between proteins and ligands. They are often an indication of the effectiveness and specificity of a ligand. Our study of hydrogen bond dynamics revealed that the complexes of BET and CCl with myeloperoxidase (MPO) were particularly stable, as indicated by the formation and maintenance of multiple hydrogen bonds during the simulation period. The observed consistency in maintaining the 1–9 hydrogen bonds in the BET complex provides convincing evidence for a reliable ligand–protein interaction. These interactions are not just static snapshots but dynamic associations that provide the complex a level of flexibility essential to functionality while providing the rigidity required for structural fidelity.

The MM-PBSA calculations enable a nuanced understanding of the binding process by quantifying the different energy contributions to the ligand–protein interaction [41]. In particular, Van der Waals energy and electrostatic energy play crucial roles in the stability of ligand-MPO complexes. The significant negative Van der Waals energies observed for CCl and BET of −112.771 ± 23.244 kJ/mol and −180.276 ± 9.119 kJ/mol, respectively, illustrate the importance of close-packed, optimal geometric conformations that control ligand affinity. Conversely, the polar solvation energy, especially for CCl, with a positive value of 253.003 ± 70.776 kJ/mol highlights the disadvantage of desolvation during binding. The resulting binding energies of −22.793 ± 30.727 kJ/mol for CCl and −140.699 ± 13.108 kJ/mol for BET possibly reflect the balance and distribution of these forces as well as the overall affinity of the ligand for MPO. The significantly lower binding energy of BET indicates its great potential as a therapeutic agent. These results not only outline the binding properties of the ligands, but also suggest the pharmacodynamic profiles required for their clinical efficacy.

Insights gained from molecular docking and dynamic simulations suggest that BET has a strong affinity for MPO, leading to stable complex formation that may translate into a noticeable therapeutic effect, particularly in alleviating MI-associated cognitive impairments. Furthermore, BET interactions with critical MPO residues have emerged as promising targets, ushering in a paradigm in which myeloperoxidase inhibition could become a crucial strategy in the treatment of post-MI inflammation and its cognitive consequences. These results highlight the potential of using anti-inflammatory agents, such as BET, in the development of nanoparticle-based drug delivery systems.

According to reports, MI increases cognitive impairment, oxidative stress, and brain inflammation [42]. The current study revealed that rats that underwent ISO-induced MI had significantly worse cognitive function, greater hippocampal and cardiac oxidative stress parameters, and lower hippocampal and cardiac oxidative antioxidant levels. These results are similar to those of a previous study [15]. Several compounds from natural sources are reported to have strong antioxidant potential and can be used as safe free radical scavengers [43,44]. The neuroprotective effect and cardioprotective effect of BET have been discussed previously regarding its antioxidative properties and anti-inflammatory activity [45,46,47]. In this study, BETNs exhibited significant cardio- and neuroprotective effects by improving cognitive function, the hippocampus, and cardiac antioxidants, similar to the effects of BET treatment on normal electrocardiograms. Furthermore, the histopathological examination of the ISO group revealed cardiac muscle fiber degeneration, cell necrosis in the hippocampal CA1 region, and significant inflammatory cell infiltration. Compared with those in the BET group, the histopathological changes in the hippocampus and cardiac myofibers induced by ISO were markedly ameliorated in the BETN plus ISO group, similar to the findings of previous studies [43,44]. The findings of this study prove that BETNs, due to their neuroprotective and cardioprotective effects, decrease ROS and MPO levels and further reduces the degeneration of the hippocampus and cardiac myofibers. The protective effects of BETNs on hippocampal myocardial damage caused by isoproterenol include their robust antioxidative effects and ability to inhibit MPO activity, as established in our investigation.

Although this study shows some promising results, it has several limitations. First, the study did not assess the long-term effects or safety profile of BETNs, an integral part of the consideration for clinical application. The molecular mechanisms underlying the therapeutic effect of BETNs remain unclear and require further biochemical and molecular studies for full elucidation. Moreover, the translation of all these findings to the clinical setting of humans from this rat model itself is challenging, as there are metabolic differences and side effects that cannot be produced with an animal model. These limitations should be addressed in future research if one is to understand and harness the full therapeutic potential of BETN.

The limitation of our study is the use of an animal model, which may not fully replicate the complexities of human physiology and pathology associated with myocardial infarction with cognitive dysfunction. Additionally, while our findings on the efficacy of β-sitosterol nanoparticles (BETNs) are promising, the long-term effects and potential side effects of BETN administration were not assessed. The study also did not explore the molecular mechanisms underlying BETN action in detail, particularly its effects on tau protein phosphorylation and β-amyloid aggregation, which are crucial for understanding its full therapeutic potential. Future studies should address these limitations by including long-term assessments and detailed molecular investigations to better translate these findings to human applications.

## 4. Materials and Methods

### 4.1. Materials

This study employed BET (SRL Pvt. Ltd., Hyderabad, India), isoproterenol (Chempure Pvt. Thiobarbituric acid (Loba Chemie Pvt. Ltd., Mumbai, Maharashtra, India), trichloroacetic acid (Thermo Fisher Scientific, Pvt, Ltd.), Mumbai, Maharashtra, India), 5,5-dithiobis-(2-nitrobenzoic acid) (DTNB) (Kemphasol Chemicals Pvt. Ltd. Vasai, India), and all reagent kits (Pariksha Biotech Pvt. Ltd. Hyderabad, Telangana, India).

### 4.2. In Silico Studies

#### 4.2.1. Molecular Docking

Molecular docking is a process in which two molecules are fitted together in a three-dimensional space, and it is a valuable tool in structural biology and computer-aided drug design. In this study, AutoDock vina (https://vina.scripps.edu/ accessed on 11 March 2024) [48] was used to carry out molecular docking. The AutoDock tool was utilized to calculate the binding of the ligand (BET) [49] to myeloperoxidase (MPO) in complex with the reversible inhibitor HX1 (PDB ID: 4C1M) [50] model, using a grid spacing of 0.375 Å and grid points in the X, Y, and Z axes set at 60 × 60 × 60. The grid center coordinates were placed at x = 17.942000, y = −19.690519, and z = −2.558926. The grid boxes were placed at the binding site of the enzyme, providing sufficient space for ligand rotation and translation. The results obtained from AutoDock were analyzed to study the binding energy and interactions.

#### 4.2.2. Molecular Dynamics

Myeloperoxidase was selected as the target in the complex with the reversible inhibitor HX1 (4C1M). BET and CCl were chosen for molecular dynamics (MD) studies. The topology of the ligand was determined using the ATB server [51]. pdb2gmx, a part of GROMACS, was used to add hydrogen atoms to the heavy atoms. The first step was to minimize the vacuum of the prepared systems using 1500 steps of steepest descent. Subsequently, the structures were solvated in a cubic periodic box with a water simple point charge (SPCE) model. Subsequently, the systems were maintained with a salt concentration of 0.15 M, and Na^+^ and Cl^−^ counterions were added in appropriate amounts to maintain charge neutrality. System preparation was based on a previously published study [52]. The simulation structure from the last 200 ns of the equilibration period in the NPT ensemble was submitted for further production runs in the NPT ensemble. The course of the simulation was followed by programs for analysis, which are integrated in Gromacs software (2024): protein root mean square deviation (RMSD), root mean square fluctuation (RMSF), radius of gyration (RG), solvent accessible surface area, and hydrogen bonding [53]. The binding free energy (BET binding), the interaction between an inhibitor and a protein, was investigated during a simulation with the function of the Molecular Mechanics/Poisson–Boltzmann Surface Area (MM-PBSA). The binding free energy was calculated using GROMACS utility g_mmpbsa [39]. We chose dt 1000 to calculate the ALO in 50 ns of the observations for the last 50 ns to obtain more reliable results.

### 4.3. Preparation of BETNs

BETNs were fabricated using the solvent evaporation technique [27]. The preparation process involved dissolving the BET in ethyl acetate to obtain 15 mg/mL, as previously reported [54]. Hexane was then added as an antisolvent to obtain a nanosuspension while maintaining a solvent-to-antisolvent ratio of 1:10. The drug nanosuspension was then prepared in a round-bottom flask connected to a rotary evaporator. The flask was partially immersed in a 40 °C water bath and a rotary evaporator at 90 rpm, and a reduced pressure of 300 mbar was used to facilitate the formation of nanoparticles upon evaporation of the solvent, which was confirmed by a nanoparticle analyzer (HORIBA India Private Ltd., New Delhi, India).

### 4.4. Animals

Healthy adult albino Wistar strain rats with body weights in the range of 150–180 g were obtained from the Vyas laboratory. Pvt. Hyderabad rats were housed under standard husbandry conditions at 25 ± 50 °C and a 12 h light/dark cycle with standard rat feed and water provided ad libitum. The experimental protocol was approved by the IAEC of the Santhiram College of Pharmacy (1519/PO/Re/S/l1/CPCSEA/2022/003).

### 4.5. Experimental Procedure

Myocardial infarction (MI) was induced by the administration of ISO (8.5 mg/kg, SC) [29] for 20 or 21 days at 24 h intervals. MI rats were divided into different groups: group I: normal control group (vehicle); group II: ISO (8.5 mg/kg, SC) for 21 days; group III: Atorvastatin (ATV) (10 mg/kg, per oral) + ISO (8.5 mg/kg, SC) for 21 days; group IV: BET (40 mg/kg, per oral) + ISO (8.5 mg/kg, SC) for 21 days; and group V: BETN (40 mg/kg, per oral) + ISO (8.5 mg/kg, SC) for 21 days. At the end of the treatment, the cognitive function of the rats was tested by the Morris water maze (MWM) test, which was employed to assess spatial learning and memory in animal models [55,56], and Cook’s pole climbing (CPC) test. The learning and memory of the animals were evaluated by assessing the conditioned avoidance response using the Cook’s pole climbing apparatus [57,58].

### 4.6. Lipid Profile Analysis

After the cognitive assessment, blood samples were collected from the retro-orbital plexus, and serum and cardiac marker levels, including CK-MB, LDH, lipid profile cholesterol, triglycerides, HDL, LDL, and VLDL, were measured using a commercial Pariksha Biotech kit (Pariksha Biotech Pvt. Ltd., Balanagar, Hyderabad).

### 4.7. Electrocardiogram Analysis

At the end of the 21st treatment, the rats were anesthetized with ketamine (60 mg/kg i.p.) and xylazine (10 mg/kg i.p.). After the anesthetized rats underwent cardiac physiology monitoring using ECG, electrodes were placed on them according to the manual method Polyrite D (Polygraph Digital, RMS, India).

### 4.8. Estimation of Tissue Parameters

At the end of the ECG evaluations, the rats were euthanized [28,59]. Brain and heart tissues were homogenized with a 10% (*w*/*v*) concentration of cold phosphate buffer (0.05 M, pH 7.4), and homogenates were then subjected to centrifugation at 1000× *g* for 10 min at 4 °C. Subsequently, the liquid portion was utilized to measure the concentrations of glutathione (GSH) [60], superoxide dismutase (SOD) [61], malondialdehyde (MDA) [62], and myeloperoxidase (MPO) [63].

### 4.9. Histological Studies

The sagittal section of the brain was incised and then kept in a 10% neutral buffered formalin solution, while the heart was similarly preserved in the same solution. The paraffin sections were prepared by embedding the brain and heart in paraffin. Sections were cut and stained with hematoxylin and eosin (H&E) for histological investigation at a thickness of 4 mm [64].

### 4.10. Statistical Methods

The mean ± standard error of the mean (SEM) was computed for all datasets in which a statistically significant difference between means was found. This determination was made by one-way analysis of variance (ANOVA) followed by a Dunnett’s comparison test. Statistical significance was set at *p* < 0.05 *, *p* < 0.01 **, and *p* < 0.001 ***.

## 5. Conclusions

In conclusion, our study demonstrated that β-sitosterol nanoparticles exhibit superior efficacy to unformulated β-sitosterol in mitigating cognitive dysfunction by reducing hippocampal oxidative damage and inflammation in myocardial infarction (MI) rats. This enhanced effectiveness of BETNs can be attributed to their nanosize, which facilitates higher concentrations at the target sites. The significant improvements observed in cognitive function as measured by the Morris water maze and Cook’s pole climbing tests highlight the potential of BETNs as a promising therapeutic approach. Furthermore, BETN administration markedly reduced the levels of oxidative stress markers, such as malondialdehyde, and increased the levels of key antioxidants, including glutathione and superoxide dismutase, in both hippocampal and cardiac tissues, reinforcing the role of BETNs in preventing oxidative damage. The substantial decrease in myeloperoxidase levels in the hippocampus and cardiac tissues underscores the anti-inflammatory properties of BETNs, which are crucial for addressing inflammation-related cognitive impairment associated with MI. Additionally, the normalization of cardiovascular markers, including lipid profiles, CkMB, and LDH, further corroborates the protective role of BETNs against MI-induced cardiac dysfunction. The restoration of normal electrocardiogram patterns and robust improvements in histological analyses of myocardial and hippocampal tissues provide compelling evidence of the therapeutic potential of BETNs in minimizing tissue necrosis and structural degeneration. While our results are based on a rat model and further studies are necessary to confirm these findings in humans, the study offers promising evidence for the use of BETNs in treating cognitive and cardiac dysfunctions related to MI. In our future research, we will focus on quantifying the phosphorylation levels of tau proteins and β-amyloid peptides, which are critical pathological markers highly expressed in the hippocampus of cognitively impaired patients. We will investigate their gene expression in cognitively impaired rats associated with myocardial infarction and evaluate the comparative effectiveness of BET and BETN treatments.

## Figures and Tables

**Table 1:** Binding energies and interaction details of BET and CCl with MPO.

Ligands	Protein	Binding Affinity, ΔG (Kcal/mol)	Amino Acids Involved and Distance (Å)		
			Hydrogen Bond Interactions	Hydrophobic Interactions	Electrostatic Interactions
BET	MPO (PDB: 4C1M)	−7.1	GLN A:91 (4.39)	PHE A:147 (6.32), ARG A:239 (4.83), PHE A:407 (6.19, 6.41)	-
CCl		−7.7	HIS A:91 (5.35), GLU A:242 (4.85), HIS A:336 (4.46)	ARG A:239 (4.30), VAL A:410 (5.13), PHE A:407 (5.44), MET A:411 (5.87), PRO A:220 (6.18 7.38), PHE A:366 (5.13, 6.21)	GLU A:242 (4.63)

**Table 2:** Binding free energies from MM-PBSA calculations of CCl and BET.

System	Van der Waal Energy	Electrostatic Energy	Polar Solvation Energy	Binding Energy
CCL	−112.771 ± 23.244 kJ/mol	−146.272 ± 27.497 kJ/mol	253.003 ± 70.776 kJ/mol	−22.793 ± 30.727 kJ/mol
BET	−180.276 ± 9.119 kJ/mol	1.010 ± 5.273 kJ/mol	61.251 ± 15.562 kJ/mol	−140.699 ± 13.108 kJ/mol

## Data Availability

Data is contained in the paper.
